# Progress of Local Health Department Planning Actions for Climate Change: Perspectives from California, USA

**DOI:** 10.3390/ijerph19137984

**Published:** 2022-06-29

**Authors:** Tisha Joseph Holmes, Ava Holt, Dorette Quintana English

**Affiliations:** 1 Department of Urban and Regional Planning, Florida State University, Tallahassee, FL 32306, USA; 2 School of Business and Industry, Florida A&M University, Tallahassee, FL 32307, USA; ava.holt@famu.edu; 3 Climate Change & Health Equity Section, Office of Health Equity, California Department of Public Health, Sacramento, CA 95814, USA; dqe1@att.net

**Keywords:** health departments, climate change policy, adaptation planning, public health, equity, barriers, California

## Abstract

Public health departments are on the frontlines of protecting vulnerable groups and working to eliminate health disparities through prevention interventions, disease surveillance and community education. Exploration of the roles national, state and local health departments (LHDs) play in advancing climate change planning and actions to protect public health is a developing arena of research. This paper presents insights from local public health departments in California, USA on how they addressed the barriers to climate adaptation planning with support from the California Department of Public Health’s Office of Health Equity Climate Change and Health Equity Section (OHE), which administers the California Building Resilience Against Climate Effects Project (CalBRACE). With support from the U.S. Centers for Disease Control and Prevention (CDC) Climate-Ready States and Cities Initiative (CRSCI), CalBRACE initiated an adaptation project to seed climate planning and actions in county health departments. In this study, we compared the barriers and strategies of twenty-two urban and rural LHDs and explored potential options for climate change adaptation in the public health framework. Using key informant interviews and document reviews, the results showed how engagement with CalBRACE’s Local Health Department Partnership on Climate Change influenced the county departments’ ability to overcome barriers to adaptation through the diversification of funding sources, the leveraging strategic collaborations, extensive public education and communication campaigns, and the development of political capital and champions. The lessons learned and recommendations from this research may provide pathways and practices for national, state and local level health departments to collaborate in developing protocols and integrating systems to respond to health-related climate change impacts, adaptation and implementation.

## 1. Introduction

Climate change is a clear and present danger to human health, placing public health departments at national, state and local scales across the globe on the frontlines of identifying and responding to the threats associated with extreme weather and other climate related events. Climate change is increasing the incidence of extreme storms, high heat days, droughts, wildfires, storms and flooding that can cause bodily injury and/or death [1,2]. Internationally, drought, heat, and windstorms are fueling wildfires that emit pollutants in the atmosphere, which can negatively impact those with respiratory problems such as asthma [3,4], displace communities, and disrupt food production and national commerce [5]. As wildfires become more frequent, and smoke transports across thousands of miles, related health risks are felt far from the source, impacting health and health jurisdictions across the globe. In the Western USA, wildfire smoke in recent years represents up to 50% of total PM2.5 exposure compared to less than 20% a decade ago [6].

Poor health outcomes can place a significant strain on the healthcare infrastructure and services, exacerbate health disparities and poverty levels, and push socio-ecological systems to tipping point thresholds [7,8,9]. Consequently, public health practitioners and institutions must address common climate threats such as wildfire and wildfire smoke, and they must develop standards for practice and response amongst competing and compounding pressures such as the COVID-19 pandemic often in a context of constrained or dwindling resources and capacity [10].

There have been calls for a greater presence of health perspectives in the climate change planning discourse [11]. The bulk of public health and climate change research focuses on identifying the incidence of current and future disease impacts of various climate hazards [12]. Attention is also directed to strategies to reduce greenhouse gas emissions with a public health benefit focus on the greening of urban areas and weatherization [13]. Yet, assessments of the approaches utilized by national, state and LHDs for planning for climate change is an understudied area of research [14,15,16].

The Climate-Ready States and Cities Initiative (CRSCI) and the Building Resilience Against Climate Effects (BRACE) framework were developed by the U.S. Centers for Disease Control and Prevention (CDC) to assist local and state level officials to build capacity to prepare states and communities for the adverse health effects of climate variability [17]. As one of the sixteen states and two cities funded by the CRSCI from 2012 through 2021, the California Department of Public Health’s (CDPH) project, California Building Resilience Against Climate Effects (CalBRACE), created the Local Health Department Partnership on Climate Change (Partnership). The partnership provided capacity-building resources, a community of practice and technical support to LHDs for planning and implementing strategies that reduce the health burden of the changing climate in both coastal and inland counties in California [18].

Since 2013, the CDPH Office of Health Equity Climate Change and Health Equity Section (OHE) has implemented the BRACE framework in conjunction with county public health departments, tribes and public agencies throughout the state. At the state level, the program coordination is intended to be collaborative and iterative in nature, seeking input from public health practitioners, epidemiological specialists and climate scientists to develop and direct technical assistance and resources to county public health departments to identify vulnerable locations/populations and implement intervention/adaptation projects [19]. As one of the most climate-vulnerable states leading advancements in implementing climate change measures, we investigate the following research questions: (1) How are public health departments in California responding to challenges faced to effectively address climate change? (2) How does the CalBRACE program influence the progress of climate and health adaptation planning? Specifically, the paper aims to assess how LHDs addressed the barriers to adaptation with empowerment, tools and other resources from the partnership in order to better inform more integrated approaches to multi-scalar and collaborative climate and health adaptation planning in USA and international contexts.

The paper begins by examining the relevant research on barriers to the implementation of climate-related adaptation into the public health framework. We introduce the partnership from 2014 to 2018 as a critical case study. Following this section, we present results from document reviews and interviews with public health practitioners on their approaches to overcoming the barriers to adaptation in local public health. We then interpret these findings and develop recommendations to inform climate and health adaptation policy and practice.

## 2. Literature Review

The connections between public health and climate adaptation research are growing in number and scope. Studies have identified the acute effects of specific climate-related events on health, chronic exposure to pollutants as well as long-term interactions between environmental factors and various health conditions occurring globally [2,20]. Research is also growing around the process of planning for climate change in the public health realm [21].

The literature identifies several barriers to climate adaptation. Studies show that a common barrier to climate adaptation in public health has been financial constraints. According to Huang et al. [22], the global management of climate change is expensive and require billions of dollars annually. Limited financial ability to support the public health sector exacerbates the impact of diseases and deaths attributed to climate change. Additionally, a lack of access to technology impedes the progress of climate adaptation actions in public health [22]. Despite the projections of global temperature increases, the uncertainty of the specific times when the impacts will occur and with what intensity, and how these will affect populations, limits the implementation of adaptation approaches. For example, floods cause deaths and health effects in different places, even in nations with adopted adaptation strategies, due to uncertainties relating to intensity projections. The uncertainties limit the preparation of public health especially in acquiring resources and funds necessary for response to emergencies [23,24,25].

In addition, climate adaptation has been influenced by the perspectives, values, processes and power structures within communities. According to research by Adger et al. [26], adaptation to climate change could be limited by social perceptions and values. For instance, societal values that have a limited concern for environmental issues could limit strategies targeting resources to ensure climate adaptation. Aylett [27] indicates that climate adaptation in public health is limited by the lack of communication and awareness on issues surrounding climate change. Populations residing in low socioeconomic areas may have limited access to information that could reduce the negative impact of extreme weather events. For example, Eisenack et al. [28] state that limited awareness about climate change impact at the local level minimizes the perception that there is a need for developing adaptation strategies among individuals.

Ekstrom and Moser [29] point to inadequacy and competition in leadership as barriers that limit climate change adaptation in public health. Eisenack et al. [28] suggest that leadership issues affect the process of decision making, which results in delays and restrictions for implementing adaptation strategies. Furthermore, Kemp et al. [30] describe that the applications of public health adaptation to climate change are limited by external politics and lobbies by various interest groups. According to the authors, managers within organizations are primarily focused on issues affecting the public directly, thus deferring efforts on policies and issues affecting them indirectly. Additionally, the political views of different leaders limit the process of developing policies that could favor climate adaptation in public health [31]. In 2013, research by Biesbroek et al. [32] stated that governments are critical players in directing climate adaptation, arguing that governments at the national, regional and local levels play a significant role in constraining, enabling and stimulating adaptation. However, limited policy guidance, lack of governmental resources, and inadequate coordination between different administrative levels are barriers to action [32].

Public health departments play key roles in predicting, responding to and protecting communities from the direct and indirect effects of increased disease and death rates because of the changing climate [33]. A survey of local health officers in California conducted prior to the development of the CDPH Climate Action Team in 2009 indicated that local health officers believed that climate change posed a serious risk to human health, but they lacked the resources and information to respond to that risk [34]. Although implementing direct and indirect climate adaptation interventions can minimize health impacts on vulnerable communities, public health departments continue to face common constraints and barriers to implementation [10]. However, the ways in which these barriers are realized and addressed differ depending on the institutional context and the internal capacity of the public health institutions [35]. Unpacking the procedural barriers encountered as well the opportunities leveraged by public health departments in planning for adapting to climate change can expand the practice of sound planning in the public health field.

## 3. Materials and Methods

We utilize the single case study approach utilizing qualitative data to gain in-depth understandings of the processes, actions and outcomes in a real-world context to inform broader understandings of climate and health adaptation planning [36,37]. We selected California as a critical case, as it is one of the most vulnerable U.S. states facing climate impacts where funding and political support for climate change action are high. California’s economic, geographic and sociodemographic size and diversity makes it a representative microcosm of spatial, environmental, socioeconomic and political variations seen across the United States. Planning for climate change has been elevated as a state priority in California. It was one of the first states to pass sweeping legislation beginning with Senate Bill 32 (SB32) in 2006, which prioritized and directed funding to climate mitigation and greenhouse gas reduction projects. Since SB32’s passage into law, there has been growing attention focused on the significance of public health outcomes and response in planning for climate change, the challenges faced and how public health departments are meeting these challenges and overcoming them [38]. The OHE CalBRACE project has participated in the BRACE program since its inception and has contributed to state and local integration of health into climate mitigation, adaptation and general plans (https://www.cdph.ca.gov/Programs/OHE/Pages/CalBRACE.aspx, accessed on 20 February 2021).

A qualitative approach consisting of key informant interviews and document reviews was used to identify and analyze information collected to understand climate adaptation processes and decision making by local public health officials engaging with the partnership. Initial contact was established with the OHE. A list of counties that engaged with the partnership was obtained and used to solicit participation into the study. Twenty-two individuals were contacted to participate from fourteen county health departments. We also interviewed officials working at the state and federal level to assess the capacity building and integrated processes across national, state and local jurisdictions. Invitations to participate were sent to participants through electronic mail detailing the scope of the study in July 2018. Follow-up calls were made if a response was not received within a week, resulting in four individuals classified as non-participants. Additional information was provided through email and interviews with CalBRACE staff. We conducted informant interviews and document reviews from public health officials in counties located in the Bay Area, Central Coast, Northern Central Valley, and Southern Central Valley regions.

### 3.1. Interviews

To capture the perception and views of study participants, a semi-structured interview protocol was developed to identify barriers of climate adaptation and to document recommendations for moving efforts forward. Questions were tailored to gain in-depth knowledge about factors that influence public health adaptations to climate change in California. The protocol was structured to obtain information about current projects, adaptation processes, barriers, resources, partnerships, communications, health equity, and lessons learned. We developed the interview tool to maintain a consistent discussion format that allowed for respondents to skip questions.

We conducted semi-structured telephone interviews with 18 informants, including county health department officials and health department administrators (N = 14) as well as state-level administrators and scientists (N = 4) engaged in climate change activities. Four counties declined to participate in interviews; however, documents related to their work with CalBRACE were obtained and reviewed. Informed consent was obtained from each participant prior to recording responses. This technique was selected to collect information from a wide range of people who had firsthand knowledge about regional and community needs. We completed interviews between August and November 2018.

### 3.2. Document Review

CalBRACE developed an inventory template tool [39] to gather and track the climate mitigation, adaptation and resilience planning activities of counties, LHDs and the public health sector as a precursor to implementing the BRACE framework. The inventory template asked LHDs to detail their climate and health activities as well as actions spearheaded by other local government agencies, community-based organizations, regional agencies and state and federal partners in order to foster local collaboration and reduce duplication of climate action efforts. Grantees were also asked to detail where and how the BRACE tools such as Vulnerability Assessments, Climate and Health Profiles, and adaption plans could be leveraged to advance climate and health. The completed inventory reporting documents were identified as robust data sources because they contain information in a standardized format from funded participants within the CalBRACE Partnership. The inventory tool is featured in the American Public Association’s 2021 online publication “Climate Change and Health Playbook: Adaptation Planning for Justice, Equity, Diversity and Inclusion”.

We reviewed completed inventories from eight LHDs which detailed the ongoing climate-related planning actions in each county. Additional reviewed documents consisted of CDPH stakeholder consultations, LHD evaluations, county/city climate and health adaptation plans, CDPH and LHD presentations and webinars, which were made available from study participants through email and/or public facing websites. Key informant interviews and documents were transcribed and coded using predetermined thematic codes. These axial codes were analyzed using qualitative analysis software NVivo 12 (QSR International, Tallahassee, FL, USA) for Windows, manufactured by QSR International, acquired via download from Florida State University Information Technology Services to identify trends, relationships and differences in the in-depth perspectives on the various interventions implemented, the role of communities in the decision-making process and the challenges faced as well as opportunities for new innovations and approaches.

## 4. Results

### Case Study: California BRACE

Partnering with fourteen counties across the state, CalBRACE created the partnership, which provided technical support, adaptation planning toolkits, resources, and guidance documents aligned with the five-step BRACE framework, guidance from the CDC Climate and Health Program, and promising practices from other CDC BRACE grantees [40]. Eleven counties received small seed grants under $10,000, and others voluntarily joined the partnership cohort’s activities. Among the resources developed with collaboration of the counties is the Climate Change and Health Vulnerability Indicators for California (CCHVIs), which includes data indicators at the census track level or at the smallest scale available to identify and prioritize where local officials should focus efforts for planning interventions for protecting public health. The CCHVIs include three domains: environmental exposures, population sensitivity, and adaptive capacity data. In 2019, the CCHVIs were visualized in an interactive online portal to assist adaptation planners with on-demand county-specific assessment data and resources for prioritizing threats, populations and interventions (https://skylab.cdph.ca.gov/CCHVIz/, accessed on 20 February 2021).

The CCHVIs include state online data resourced from Cal-Adapt, which provides scenarios of how climate change may impact California, climate data, and adaptation plan development. After assessing local climate threats and vulnerabilities and projecting the most relevant health impacts of climate change, then action can be taken by LHDs to identify, prioritize and incorporate climate adaptation strategies and interventions for public health. To accelerate the planning process, building on lessons learned from the partnership, CalBRACE continues to provide technical assistance, and it curates and shares planning and action strategies specific to California primarily, and those used by LHDs and others to build capacity and increase community resilience in the California Department of Public Health’s Adaptation Planning Toolkit (https://cdphdata.maps.arcgis.com/apps/MapSeries/index.html?appid=4093397556b4450ea563f23fcf353c64, accessed on 6 June 2022).

We examined the LHD inventory reports on climate and resilience plans in public health/health sector planning activity. The LHDs reports identified plans from their local areas and public health departments or health sectors that address or could be enhanced to address their climate adaptation activities, and specific areas of plan content and gaps. Eight LHDs identified fourteen plans at the city, county, regional and federal levels in addition to plans developed by universities between 2014 and 2016. The review of the inventories revealed that health, adaptation and health equity are included in regional-level and university partner plans. However, the focus on health content was limited at the city scale, since most health department jurisdictions are at the county level. While health and climate adaptation measures were considered to some extent, at the county level, there was no explicit focus on issues related to health equity. One federal plan was identified as having relevance to climate change planning; however, it had no references to health, climate adaptation and/or health equity (Table 1).

The approaches identified by LHDs as spotlights of current local action to respond to climate change that were most frequently identified in the inventories were implementing emergency response interventions such as cooling shelters during extreme heat episodes, evacuations during wildfires and provision of potable water during drought periods (5), updating emergency or heat response plans (4) and conducting public education/communication campaigns (3). Interviews conducted with LHDs two years after the inventories were developed; these revealed that LHDs had pursued a broad range of activities including the development of localized vulnerability assessments and projecting disease burdens (12/14), participation in working groups and collaborations (12/14), formation of advisory committees (9/14) and communication and public awareness campaigns (9/14), creating toolkits and/or health and hazard profile reports (5/14, respectively). The least cited approaches to expanding activities were the pursuit of additional grant funding (2/14) and the development and implementation of comprehensive climate and health adaptation plans (1/14) (Table 2). All inventories and interviews acknowledged a need to focus on equity implications for marginalized/frontline and environmental justice seeking groups who would be disproportionately impacted by the effects of climate change.

The inventories also examined the needs and challenges that LHDs faced while planning for climate change. Funding was the most prominent need reported by LHDs (5/8). Specifically, LHDs cited that having access to a sustained and large pot of financial resources would help expand the scope, type and scale of actions pursued. Increased funding was also regarded as a means of alleviating other existing barriers such as increasing organizational capacity, dedicated staff and full-time employees instead of those who are often serving on multiple grants/programs and priorities. Lack of funding and resources and competing priorities were most often cited as the most significant barriers in the interviews as well. Of the twelve counties interviewed, all expressed that funding was a primary barrier to the implementation of climate adaptation initiatives. Respondents from all regions indicated the same sentiments that underlined the significant constraints limited resources placed on their ability to prioritize climate action while balancing other competing demands, which in turn inhibited their ability to secure more funding to advance ongoing efforts:

“We didn’t have any money. We all just kind of carved the time out of our existing workloads. None of us have funds to do the work”.

“We need more funding, but we also need assistance with grant writing to secure funding”.

A related issue revolved around the lack of climate change expertise and capacity. Officials in smaller counties shared the sentiment of one interviewee who remarked that while public health departments were in a good position to collect and analyze local health data, the focus and expertise required to understand the influences of climate change was uncharted territory. In another county, staff thought that the lack of climate change expertise, coupled with the lack of financial and human resources, created a significant burden on staff time, which then had to be “pieced together and often done inconsistently”, since staff members are committed to work on other grant-funded programs. One Central Valley health department official explains:

“We often feel stretched in terms of our workload and giving the assignment of trying to drive climate change activities is a difficult task especially when it’s a touchy subject… While we didn’t need a whole lot, to obligate us to start doing something long-term we will need some funding to help us drive that forward”.

Most of the smaller and more rural counties in the Central Valley noted that the invitation from a state agency to participate in the CalBRACE program enabled them to justify the focus on climate and health issues. Additionally, CalBRACE was the sole source of funding to support climate and health work, or these counties would not have been able to pursue any climate adaptation initiatives. Some counties interviewed sought to expand and diversify funding support by procuring additional grants to support their work. For example, a county in the Bay region that actively searches and applies for grant funding stated:

“….one thing I’ve found is that in California, [there] are a lot of grant opportunities and that’s why we’ve been successful in our assessment…I found bringing in grants—is a great way to expand the work and show the value and then I found leadership to come on board”.

One Central California area county’s work focused heavily on health equity and disaster preparedness, which was supported entirely by grant funding. While this enabled the unit to align their efforts to potentially pursue longer range climate adaptation work, having to adhere to grant scope requirements constrained their ability to work beyond disaster response projects that did not directly relate to the grant:

“We are doing the public health emergency preparedness to build community resilience and identify hazards, but that’s more of the immediate and not long-range. If that was in our work plan then we could do it, but it’s not…. Right now our frame for health equity, we’re trying to implement things that our community prioritized, things like vulnerable chronic diseases. So it would be hard to take resources away from those areas and reshift them to climate adaptation. Even though climate adaptation does have an impact on vulnerable populations”.

The public health official went on to highlight the other difficulty of aligning time frames and lenses between emergency preparedness and climate adaptation planning orientations:

“I know you’re talking about things that might happen in the future. She’s just talking about emergency planning in general. I’m talking about things that are happening right now and whether they have food security today and not 10 years from now”.

The official suggests that stronger directives from leadership to integrate climate into existing emergency preparedness workplans would be an effective way to start mainstreaming climate change lenses. A natural pathway to mainstreaming could reside in divisions that already have expertise in community health and equity, since all county health departments are mandated to have emergency preparedness and response programs that specifically target the needs of at-risk populations.

Promoting more engagement with relevant stakeholders was also identified as a high priority need. While several agencies at the local, regional and state level were identified (Table 3) as working on climate change, inventories indicated there was a greater need for collaboration and the integration of health perspectives in the planning, coordination and implementation for climate-related plans and activities (6/8).

Public health officials in the Bay-Delta region indicated through interviews that identifying linkages between climate adaptation and environmental programs began with projects focused on the built environment. Establishing relationships with local government agencies and community partners was documented as a key component toward working around barriers to implementation. Thus, implementing climate-related projects was supported by partners with similar goals. According to interviewees, these collaborative efforts brought in expertise across multiple disciplines, agencies and industries that increased communications and trust:

“I was working on built environment issues, planning issues, working with the county planners and food systems, all these things that kind of linked up to climate change”.

“In collaboration with the Public Health Department Emergency Preparedness program, Epidemiology section, County Office of Emergency Preparedness, and other Public Health partners, the Heat Response Plan originally drafted in 2008 will be updated to recognize and incorporate climate change effects. This includes ensuring that Cooling Centers and other mitigation efforts are appropriate to meet the community needs. Collaboration in these efforts will include the homeless task force, farmworker coalition, Area Agency on Aging, and other organizations and agencies that serve vulnerable populations”.

In addition, building and leveraging multiple sectors around climate and health-related work gave rise to informative research. Participants indicated that studies around climate and health-related issues provided the needed scientific data to inform decision making and to address local priorities. For example, counties in the Bay and Bay-Delta region provided the following:

“The first thing we did was a health co-benefits analysis on our county’s climate action plan. So, for every mitigation measure they adopted that would reduce greenhouse gas emissions, we analyzed the health co-benefits of that measure to show that there are health benefits [in] some measures and some more than others. And gave policy makers more information [about] which measures they ought to fund and which may not have health co-benefits”.

“The health department runs the Emergency Operations Center during heat events, our public health emergency response branch, so we work very closely with our public health emergency preparedness response in developing procedures for extreme heat and response planning”.

From the interviews and examination of the inventory reports, creating partnerships with multiple agencies, local community partners, and organizations was a key factor in advancing planning climate adaptation goals. The collaborations joined people with similar goals toward achieving sustainable communities and understanding challenges from different perspectives. For example, the Bay Area and Bay-Delta Regions acknowledged working closely with planning departments, public utilities, and emergency preparedness in developing an extreme heat index for emergency response. Another example included the formation of a Sea-Level Rise Working Group that works toward addressing coastal flooding and implementing climate adaptation strategies. Respondents indicated that the benefits of creating collaborations included building capacity, leveraging resources, and understanding the linkages between programs. For example, one of the counties located in the Bay-Delta region stated:

“Being associated with the Capital Region Climate Readiness Collaborative, (CRC), where that organization is one of eight different regional collaboratives under the umbrella of a statewide alliance of regional climate collaboratives of California. The work that I was doing [was] from the standpoint of we can’t be doing the work that we’re doing right, if we don’t consider a lot of the factors that the BRACE framework addresses. So, we started building and then all just signed on and then started leveraging multiple sectors in and around doing climate change and health-related work”.

In some cases, integrating the health component into existing climate action efforts was made on “as possible” basis; however, disciplinary dominance and gaps remained. As one health department official indicated:

“The City planning division [was] already doing work on their climate action plan. They had several meetings. I came in in the middle when they were doing their plan and it was a lot about planning so, I tried to inject the health plan wherever. It was mostly just planning, so half the jargon I didn’t understand, but they were strong partners”.

A third category of needs is related to developing technical capacity to effectively assess vulnerability, project disease burden and utilize locally relevant analytical tools (4/8). Two inventories specified the potential utility of centralizing assessment tools and methodologies in a resource center for LHDs. The sharing of data, methodologies and approaches can create opportunities for learning, exchange and the diffusion of ideas around integrating existing epidemiological expertise with climate science and projections.

To make informed decisions, public health officials indicated the need for supportive data that assesses risk as an essential component in directing resources and prioritizing local public health issues. Respondents provided insight into how counties assessed climate change impact on vulnerable populations. By identifying communities that are most susceptible to the health impacts of climate change, public health officials can target these areas for tailored intervention strategies to reduce health impacts. To better understand regional needs, data collection from vulnerability assessments and climate reports provide information to inform decision making. For example, public officials in the Southeast Sierra and Bay-Delta regions conducted a preliminary review of existing measurements of temperature data and forecasted projections of the potential rise in temperature impact on residents. Various regional collaboratives conducted economic impact studies and health co-benefits analysis that provides the data to inform communities about the potential effects of climate-related hazards.

Scientifically assessing the impact of climate change on health provides the evidence required to assess potential risks and to inform policies. The findings from vulnerability assessments, climate studies, and climate-related research identify potential climate impacts on human health at the local scale. For example, one county official reported that a Drought Task Force, consisting of multiple county departments, coordinated efforts for monitoring, conducting impact assessments and responding to emergencies at the local level. The task force findings serve to inform the public about potential health hazards, to provide explanations for impacts and to identify specific local areas at higher risks. From the assessment outcomes, communities can rank priorities of concern. Despite these advancements, the equity connections between climate and health are still not clear in some communities. As one Central Valley official describes that while vulnerability indicators are available in the context of climate vulnerability:

“It hasn’t been recognized as a disparity. It’s been said that climate is affecting everybody, the same sun shines on everybody”.

A final category of identified challenges underscored the role education and communication could potentially play in garnering broader political support, commitment and agency in developing stronger public policies and mandates to address the health impacts related to climate change through strategic updates and implementation of plans and policies. Public health officials pointed out the need for both public and political support to implement climate-related strategies that can weather changes over time. While awareness of climate-related events and increasing scarcity of water occurring across the state cannot be denied, in some places, there still is hesitation about attribution to the phenomena of climate change and taking action to solve the problem. One locality that had much success with public education and outreach initiatives under the purview of a long serving administrator who was concerned about climate change developed a climate and health profile report to better communicate the links between climate and health in layman’s terms. However, with a change in administration soon after the profile’s development, the county’s involvement in CalBRACE ended, and the efforts were relegated to the “sidelines”, since climate change was no longer a priority, and challenging this change would not be in their best political interest, even if ongoing droughts would devastate the majority of the county’s agriculture-based economy:

“That’s pretty much where we’re at now. At this point we’re not doing anything towards climate change. California is not monolithic. Coastal areas tend to be more blue and the central areas tend to be more red. The political reality is different here than it is on the coast. I think it’s a reflection of that. That’s kind of where we are at”.

Public health officials also reported that they changed the narrative from a climate change issue to a public health problem impacting vulnerable populations. Some counties were very inhibited by local political contexts that prohibited acknowledgement or discussion of climate change as a “red flag” issue. This led one public health official to withdraw from the project, and others used coded language such as “extreme weather”, “changing climate” and specific climate threats such as wildfire, drought, or heat to communicate with partners. Some public health officials see the matter as an issue of framing and focusing public perception. In order to move forward, it strategically makes sense to focus on preparedness and adaptation rather than the underlying drivers:

“It’s easier for me to jump onboard with climate adaptation than climate change. You think climate change, people are like, what? They start thinking about global warming and is it our fault? And whose fault is it? And we move the conversation over to adaptation: This is happening. We know this is happening. How do we adapt? We don’t address why it’s happening, we just address how to adapt. It’s an easier conversation”.

By using more palatable language that was less politically charged, problems can be addressed without broaching “hot button terminology that gets people defensive”. In one county, the lack of political buy-in may have influenced the ways in which the LHD tried to communicate the urgency of the issue, which further reduced interest and participation:

“We weren’t able to get any interest from our public health emergency advisory committee, or any of our local health systems. It was a little bit sad. I remember the presentation which really went against what we know now as a positive way of communicating climate change areas. A lot of gloom and doom which is not how people want to hear about climate change. We have not had a lot of positive experiences with trying to communicate the urgency to our partners… we haven’t found a communication strategy to really raise awareness and engage partners, community residents, on the issue. How do you tell people they need to do something without scaring them too much, but just enough so they feel compelled to take action? I think that’s what we’re still struggling with”.

However, due to the subsequent catastrophic wildfires, heat waves and droughts of the recent years, all counties are no longer prohibiting county employees from discussing or planning to reduce harm from climate change.

## 5. Discussion

Planning for climate change is a multifaceted process requiring the sustained engagement of relevant actors, including communities, and it is informed by the best available data and science to understand local vulnerabilities and risks, to make evidence-based assessments of strategies and to implement integrated plans, policies and programs [41,42]. Across all the funded LHDs examined, the CalBRACE Partnership expanded the existing technical and financial capacity of county health departments to pursue climate adaptation planning. This support went a longer way with some than others. The most successful health departments were well-funded, high-capacity agencies well positioned to leverage the support provided by CalBRACE. The additional funding provided those without dedicated staff a foundational structure to guide the process of taking climate change into account and to respond by developing new projects and enhancing or expanding existing programs.

This study revealed the regional differences in the hazards of concern, which were closely tied to geographic and demographic features of the communities. Most health departments utilized elements of the BRACE framework to guide their process of setting goals, developing strategies and formulating plans. The majority of health departments focused on refining surveillance intelligence to better inform policies, updating educational and outreach materials and producing information for public consumption related to the risks facing communities within their counties. There appeared to be a distinct difference in the orientation of more urbanized coastal counties compared with more inland and rural counties.

The literature on climate adaptation planning is biased to more wealthy urban contexts compared with smaller towns and rural areas; however, the dichotomy may be inconsequential in the context of the scale of change needed. The coastal counties focused on a broader range of hazards, regional vulnerability assessments and implemented new pilot projects while developing stronger collaborative partnerships. The central, inland and rural counties focused more on responding to a lack of water, with a focus on economic impacts, specific vulnerable outdoor worker groups and updating existing programs. These regional differences go beyond simple geographic factors, but they also reveal the differences in communicating what interventions and actions are possible when responding to climate change [43]. Similar to other environmental studies in more conservative contexts [44], we see public health officials framing the issue around the specific environmental hazard rather than drivers or causes of climate change [45]. Additionally, the focus on the economic dimensions of the outcomes of climate change resonate with business leaders and decision makers who hold significant political clout [46]. Framing the climate and health adaptation narrative can remove the spotlight from the urgency of mitigation to reduce further global warming [47]. Yet, by finding multiple ways to communicate the risks and options for adaptation to the public and various decision makers, public health professionals are starting to build a discursive space for audiences to interpret, shape and reframe the space climate change occupies in relation to health narratives and perspectives.

A recent survey of California health departments and subject matter experts conducted by Bay Area Regional Health Inequities Initiative (BARHII) underscored the need to “elevate climate resilience and health equity planning, and implementation in LHDs and to ensure appropriate (not simply adequate) fiscal resources and technical assistance aligned with its import and effects on Californians” [48]. While respondents all emphasized the urgency related to developing a sustainable governance structure for LHDs to address the impacts of climate and health, particularly in frontline communities, existing programs and levels of resources and support are not adequate to effectively respond to the scale of the problem. These challenges are well documented in the public health management sphere, and research has indicated that in the absence of these significant barriers, practitioners may be able to pursue more progressive actions [49]. However, the narratives emerging from LHDs indicate more nuances to the importance of having champions to strategically leverage relationships and find opportunities for mainstreaming climate adaptation actions in existing and new public health programs. The conditions are most favorable for success and are enhanced by a supportive organizational culture where the value of the work on climate adaptation is explicitly acknowledged and supported from the top down and the bottom up [33,50].

Synergies between leadership and commitment to the cause and the process served as key motivations behind the actions of program managers [51]. Part of the commitment is embedded in the structure of receiving funding and having compliance in reporting requirements on activities conducted. This procedural form of commitment, while important for ensuring deliverables are generated according to desired timelines, would engender credibility when standardized projects met the minimum requirements, such as developing the inventory reports in the CalBRACE Partnership. The urgency behind the current and future impacts of climate change on vulnerable populations pushed these local officials to clearly identify the risks posed and develop targeted strategies even in the absence of internal guidance or institutional support. Another factor that was/is absent is the support of a robust institutionalized field of practice with established resources, infrastructure and substantive evidence-based practices, which exist in public health disciplines or initiatives such as chronic disease, maternal child and adolescent health programs and environmental health [52]. Establishing infrastructure, practices and resources for climate adaptation in public health requires building out and institutionalizing infrastructure and resources while simultaneously doing the adaptation work as well. None of these counties seemed to have an established infrastructure for their work on climate change, and as stated, they worked on this in the margins of their other duties as time allowed. The ideological commitment to protecting public health above all else appeared to give administrators the flexibility to dedicate resources and attention to addressing climate risks in the context of existing frameworks, policies and plans with limited political liability. This commitment at the local level, when bolstered by institutional support from the top national and state levels down, powered the momentum for local officials to elevate and innovate beyond the traditional boundaries of the existing public mandate [53].

Collaboration within the health department and external partners emerged as a common and effective strategy for health departments to advance their climate adaptation and preparedness measures. There is growing research in the field of climate adaptation on the role of intermediary organizations working as collaborators and connectors to increase cooperation among actors, the diffusion of science and information to stakeholders and creating spaces for idea generation and innovation [54,55]. These boundary-spanning organizations [56] operate as junctures that expand the capacity for local actors to evaluate their community vulnerabilities, develop targeted strategies, garner political support and implement strategies in a sustained and mainstreamed fashion [57]. As seen in the Santa Clara County collaborative, existing connections with the Office of Sustainability and Planning enabled the health department officials to align their planning efforts in a unique way that generated multiple benefits and spread the administrative responsibility across a broader swath of actors. Multiple counties were successful, leveraging local collaborations to acquire funding for climate adaptation planning and work in recent years. Regional coalitions such as the BARHII and the Alliance of Regional Collaboratives for Climate Adaptation (ARCCA), similarly to the climate compacts in Florida, offer collaborative platforms to develop communities of practice, share knowledge and exchange ideas [35]. The value of an interdisciplinary collaboration with multiple disciplinary expertise and resources cannot be understated as a crucial factor for cross-sectoral mainstreaming and building sustained partnerships. This finding aligns with the need to reverse the segmentation of climate resilience work across jurisdictions and promote the multi-sectoral integration of partners and operations during planning, response and implementation phases as detailed in the recent survey of needs and priorities of LHDs conducted by BARHII. However, the survey also notes that the participation of LHDs and integration of the value of their experience with addressing health equity issues, utilizing community level knowledge and building trust with community partners are overlooked in community-wide resilience planning processes initiated by other sectors of government. While disciplinary differences in terminology, timelines and approaches may generate some misalignment, even conflict, within overall goals, using a justice, equity and inclusion orientation that emphasizes deliberation, commonalities and aligned multiple benefits can set the stage for more effective collaboration [58].

Uncertainty remains around the sustainability of action should the funding spigots run dry. Embedding long-term changes that promote more robust climate resilient actions requires consistent and sustained implementation, evaluation and improvements [59]. However, the limited funding associated with pilot projects, while generating targeted support to spur innovation, can amount to a short-term initiative with a few measurable outcomes to indicate progress. Scaling local climate and health actions to a city or county-wide level also presents a challenge without commitment to a broader, dedicated program, resources and technical capacity to support the expansion. BARHII survey respondents indicated that a modest increase in financial support would need to be adequate for dedicated staff positions to sustain the effort [48]. In the United States, with bills such as the Build Back Better stalled in Congress, federal and state agencies rely on the same funding sources to sustain and to expand existing climate and health initiatives, which portends to be an ongoing and unsustainable constraint as the health impacts of climate change and the urgency for action grow. Reliance on piecing together overburdened and episodic grants can leave health departments vulnerable to shifts in allocations and political priorities and personnel, and progress in climate and health work can stagnate or disappear altogether.

In an effort to link national and state resources to local health departments initiated through the partnership activities and seed funding for climate and health adaptation planning, public health officials faced challenges, seized opportunities and are gradually gaining ground in California. The California Legislative Version of the 22–23 State Budget includes an initial $25 million investment ($1.25 million in State Operations and $23.75 million in Local Assistance) in one-time General Fund expenditure authority at CDPH to establish a Climate Change and Health Resilience Planning Grant Program that funds local health departments, community-based organizations, and tribes to develop regional Climate and Health Resilience Plans. Additionally, 30 positions and $10 million of the General Fund were approved in 2022–2023 and ongoing at CDPH to initiate Climate Change and Health Surveillance to provide near real-time notification for public health departments, first responders, and the community for emerging or intensified climate-sensitive diseases [60].

Through its CRSCI and the BRACE program, the CDC set goals for developing state and local climate and health adaptation plans. The partnership made this linkage viable and supported local public health officials in building their regional and local networks for initiating and coordinating adaptation practices and increasing their capacity for a public health system response to climate related health threats. Through national, state and local systems and collaborations, local public health practitioners were able to both contribute to and benefit from an emerging learning community of practice, furthering the field of climate and health in the United States. Public health practitioners in California that participated in the partnership were empowered and eager to codevelop and master professional competencies to build community resilience in public health systems and the populations they serve. Most participants developed strategies to overcome barriers and move forward with planning steps and activities tailored to their communities. Some were overtaken by competing public health priorities, decreased staffing, and local political environments and found it difficult to add climate work into an already full workload. With coordinated linkage between national, state and local health departments, ever greater systems and community level resilience can be fostered through a network of multi-decadal, diverse and reliable funding sources to support climate-related professional development, surveillance, data analysis, project implementation and evaluation of outcomes in diverse communities and geographies, such as California. These integrated networks for resilience in the public health and hospital systems and communities should include federal, state and local government networks, resources and investments matched by and enhanced by the academic, philanthropic and private sectors.

## 6. Conclusions

While the impacts of climate change pose significant health challenges to populations, efforts to implement climate adaptation into the public health sector have been challenged by barriers that include lack of resources, financial difficulties, conflicting priorities and limited leadership/government involvements. We suggest the utilization of strategies that include building the capacity of national, state and local health departments through technical and funding support, finding congruence to expand and enhance existing programs, promoting diverse and strategic forms of communication to engage different audiences, usage of commitment from inspired leadership and staff, and promoting collaborative partnerships and interventions to ensure mainstreaming across sectors. This illustrative case study also raises the ongoing questions on the position and roles that public health professionals will play as agents of implementation to advance progressive climate action in national, state and local public health systems and other sectors. Climate change can no longer be just a peripheral issue for existing public health programs or a corollary addition to ongoing resilience initiatives, as increasing changes in temperatures, droughts and wildfires become public health threats internationally. Public health and hospital systems are central to planning for their own roles and resilience, and the preparedness of communities, healthcare infrastructure and healthcare professionals during critical events. The climate crisis already has diverse and severe effects requiring public health and health systems to prepare response plans tailored to local characteristics of geography, predicted weather and indirect impacts, population, and potential for compounding events occurring simultaneously [61].

The key role national and state agencies play through administrative mandates, as sources of technical and fiscal capacity and advocates for action, is enhanced with LHD participation in the California BRACE program. Yet, the ability of LHDs to digest and translate these ranges of resources in their daily work is constrained by the very barriers that limit progress. This case study indicates the need for a more intensive focus on shifting from a reactive model to a proactive orientation to train public health professionals on how to identify and collaborate with established climate practitioners and sectors, and leverage internal and external resources to plan, evaluate, prevent and respond to climate-related health threats. Additionally, predicted climate change impacts and health threats should be explicitly included in national, state and LHD strategic plans and population and community health assessments. Health department leaders must elevate climate change as a priority focus area that is embedded and mainstream across their departments and programs. While the lessons from the California case are illustrative, local and regional contexts across states and cities can also generate variations in how the process of climate and health adaptation planning occurs. This case study sets an initial foundation to compare planning actions across BRACE grantees (forthcoming). Further examination is also needed to understand how national and state-level guidance can be moderated by LHDs and the role regional boundary organizations may play in facilitating climate adaptation actions.

## Figures and Tables

**Table 1:** Compilation of plans reviewed by LHDs and content related to health, climate adaptation and health equity.

Jurisdiction	Number of Plans	Health Content	Climate Adaptation	Health Equity
City	4	0	2	0
County	5	2	2	0
Regional	2	2	1	2
University	2	1	2	1
Federal	1	0	0	0
**Total plans**	**14**	**14**	**14**	**14**

**Table 2:** Planning activities used by 14 LHDs to respond to climate hazards.

Planning Activities	Number of LHDs
Vulnerability studies/indices	14
Working groups/collaborations	12
Communications/public awareness campaigns	10
Advisory committees	9
Health profiles/Hazard reports	5
Toolkits	5
Seeking Grants	2
Comprehensive Adaptation Plans	1

**Table 3:** Relevant/Potential Stakeholders Identified by Local Health Departments (LHDs) in template.

Stakeholders Identified	Number of LHDs
University	3
City Planning Department	7
Emergency Management	5
County Planning/Government	8
State Government	2
County Commission	1
Research Institute/Non-Profit	2
Federal Agency	1
Regional Collaborative/Government	4
Social Services	2
